# Supplementary material for: WRN inhibition leads to its chromatin-associated degradation via the PIAS4-RNF4-p97/VCP axis
Source: Nat Commun. 2024 Jul 18;15:6059. doi: 10.1038/s41467-024-50178-3 (PMC11258360; doi:10.1038/s41467-024-50178-3)
Supplement: Supplementary file 1 — Supplementary Figs. [file 41467_2024_50178_MOESM1_ESM.pdf]

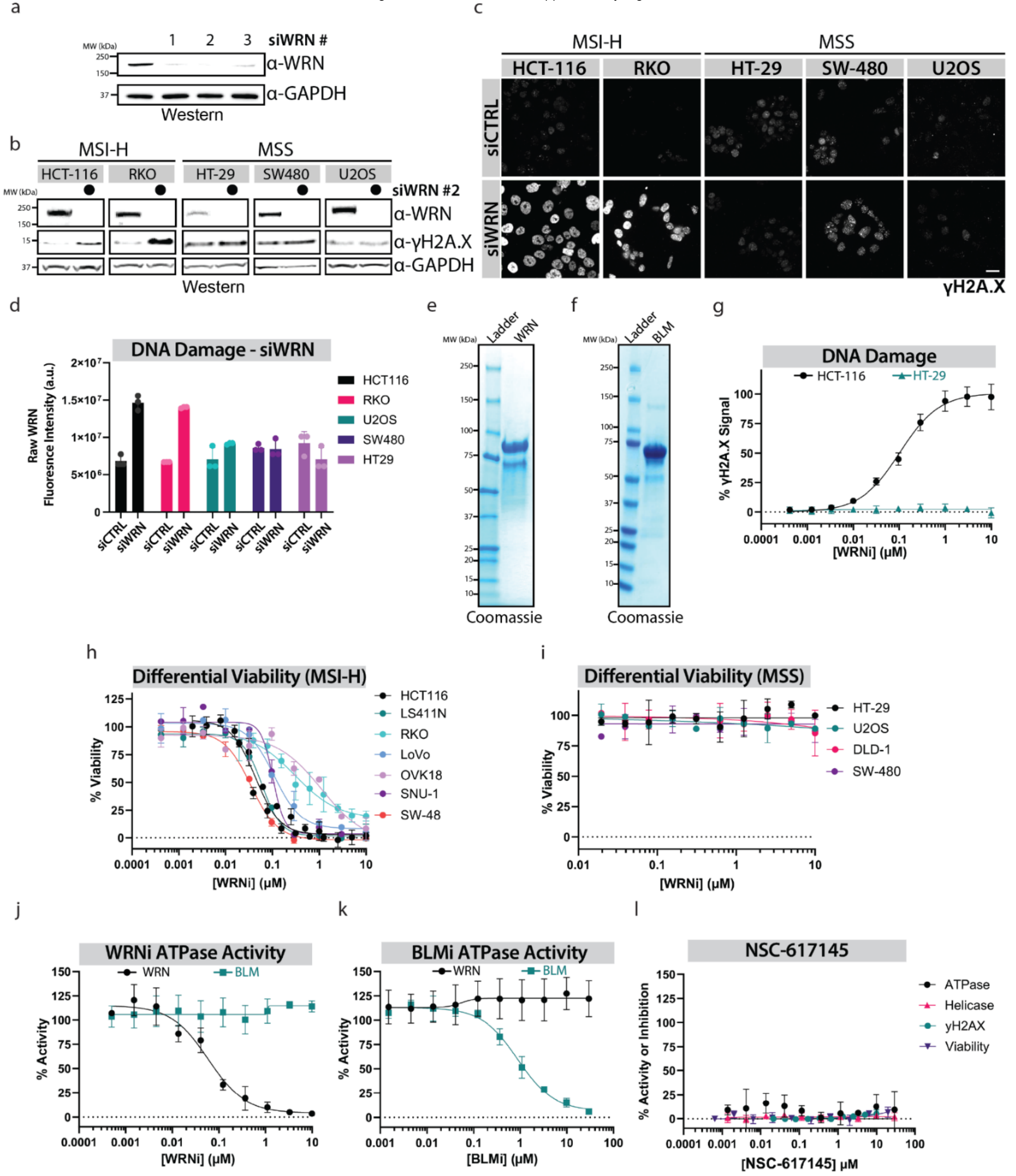
a
MW (kDa)
1 2 3 siWRN #
250
150
α-WRN
37
α-GAPDH
Western
b
MSI-H
MSS
HCT-116
RKO
HT-29
SW480
U2OS
siWRN #2
MW (kDa)
α-WRN
α-γH2A.X
α-GAPDH
Western
c
MSI-H
MSS
HCT-116
RKO
HT-29
SW-480
U2OS
siCTRL
siWRN
γH2A.X
d
DNA Damage - siWRN
Raw WRN Fluoresnce Intensity (a.u.)
HCT116
RKO
U2OS
SW480
HT29
siCTRL
siWRN
e
MW (kDa)
Ladder
WRN
Coomassie
f
Ladder
BLM
Coomassie
g
DNA Damage
HCT-116
HT-29
% γH2A.X Signal
[WRNi] (μM)
h
Differential Viability (MSI-H)
HCT116
LS411N
RKO
LoVo
OVK18
SNU-1
SW-48
% Viability
[WRNi] (μM)
i
Differential Viability (MSS)
HT-29
U2OS
DLD-1
SW-480
% Viability
[WRNi] (μM)
j
WRNi ATPase Activity
WRN
BLM
% Activity
[WRNi] (μM)
k
BLMi ATPase Activity
WRN
BLM
% Activity
[BLMi] (μM)
l
NSC-617145
ATPase
Helicase
yH2AX
Viability
% Activity or Inhibition
[NSC-617145] μM

Supplementary Fig. 1:

a. Whole cell lysates of HCT-116 after siRNA depletions of WRN for 24 h and probing with a WRN antibody show a robust loss of WRN protein. **b**. WRN depletion is synthetic lethal in MSI-H cells but not MSS cells. Whole cell lysates in the indicated cell lines after treatment with siWRN or siCTRL oligos for 48 h, and subsequently analyzed for DNA damage induction by Western blot. **c**. As in **b**, but cells were fixed in paraformaldehyde after siRNA treatments, and DNA damage was measured by measuring γH2A.X levels via immunofluorescence. Scale bar = 20 µm. **d**. Quantifications of **c.** Each graph represents the mean of *n* = 3 plates. **e**. Purification of WRN protein from SF9 insect cells. Coomassie gel staining shows a product of the expected protein molecular weight after purification. **f**. Protein purification of BLM protein from *E. coli*. Coomassie gel staining shows a product of the expected protein molecular weight after purification. **g**. DNA damage induction in MSI-H cells after WRN inhibition is dose-dependent. Dose response curves measuring DNA damage response via γH2A.X levels in HCT-116 cells or HT-29 cells after treatment with WRNi for 24 h. Graphs represent averages from *n* = 6 plates. **h**. Cell viability panel of MSI-H cells showing the differential viability effect of WRN inhibition towards MSI-H cells. Dose response curves measuring the viability of the indicated cell lines after WRNi treatment for 5 days. Graphs represent averages from *n* = 3 plates. **i**. Cell viability panel of MSS cells, performed as in **h**. **j.** Dose response curves measuring the *in vitro* ATPase activity of WRN or BLM after WRNi treatment. Graphs represent averages from *n* = 6 plates. **k.** Purified BLM protein is active. Benchmarking of BLM protein by treatment with BLMi. Dose responses measuring ATPase and helicase inhibition by BLMi. **l.** Characterization of the previously reported “WRN inhibitor” NSC-6174145. This compound is inactive across all assays tested. All curve fits were done by fitting a 4-parameter logarithmic regression curve. All error bars represent standard deviation (s.d.). DMSO is dimethyl sulfoxide; WRNi is HRO761; BLMi is the BLM inhibitor Compound 2. MW is molecular weight. For all WBs, GAPDH was used as a loading control.

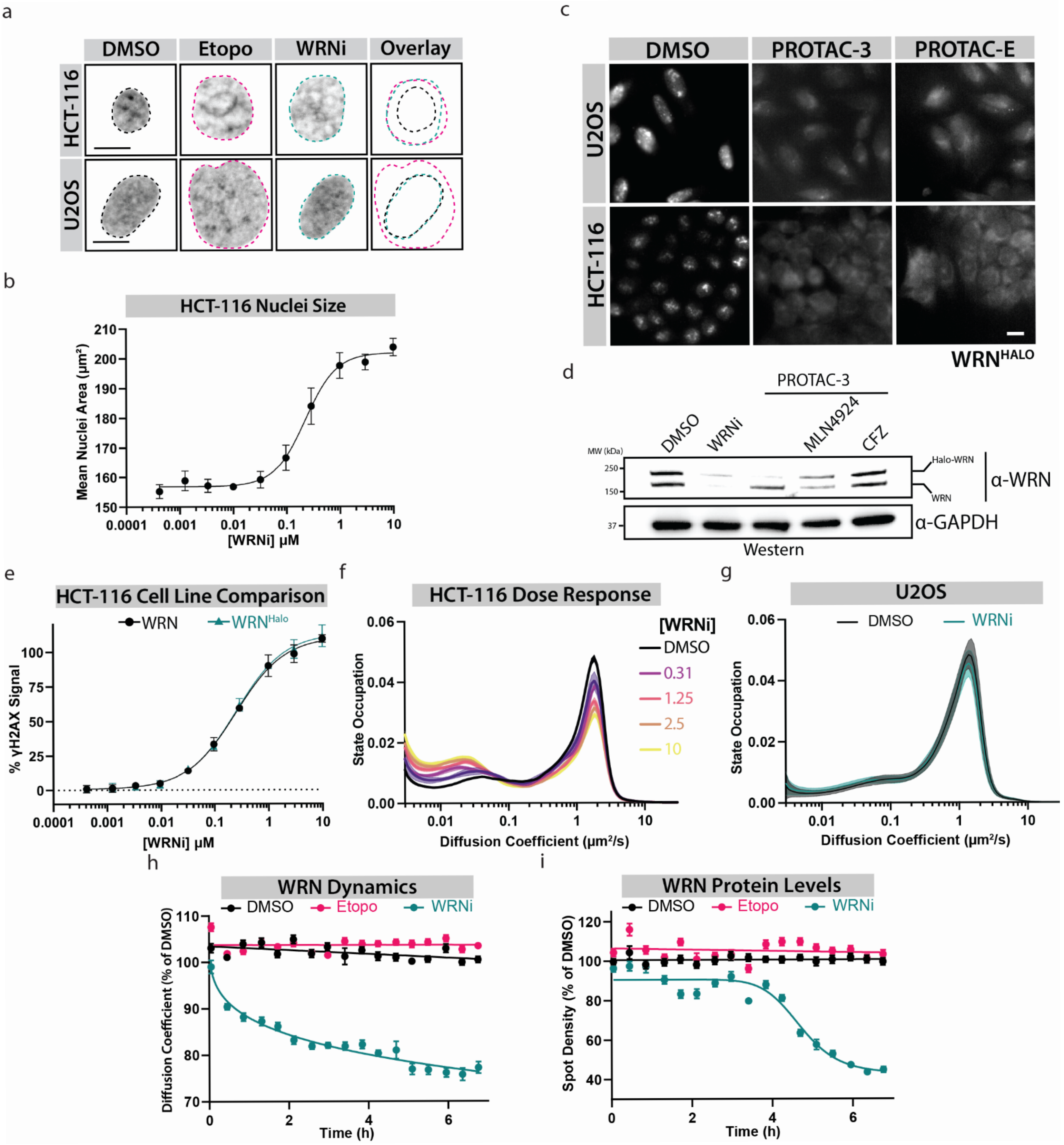
a
DMSO
Etopo
WRNi
Overlay
HCT-116
U2OS
b
HCT-116 Nuclei Size
Mean Nuclei Area (µm²)
[WRNi] µM
c
DMSO
PROTAC-3
PROTAC-E
U2OS
HCT-116
WRN^HALO
d
PROTAC-3
DMSO
WRNi
MLN4924
CFZ
MW (kDa)
250
150
37
Halo-WRN
WRN
α-WRN
α-GAPDH
Western
e
HCT-116 Cell Line Comparison
WRN
WRN^Halo
% γH2AX Signal
[WRNi] µM
f
HCT-116 Dose Response
[WRNi]
DMSO
0.31
1.25
2.5
10
State Occupation
Diffusion Coefficient (µm²/s)
g
U2OS
DMSO
WRNi
State Occupation
Diffusion Coefficient (µm²/s)
h
WRN Dynamics
DMSO
Etopo
WRNi
Diffusion Coefficient (% of DMSO)
Time (h)
i
WRN Protein Levels
DMSO
Etopo
WRNi
Spot Density (% of DMSO)
Time (h)

Supplementary Fig. 2:

a. WRNi causes morphological changes to nuclei due to DNA damage accumulation in MSI-H cells. Images of Hoechst-stained nuclei of HCT-116 or U2OS cells treated with 10 µM WRNi, etoposide, or with DMSO (vehicle control) for 24 h. Nuclei outlines are overlaid, showing the large change in area after WRNi treatment in HCT-116 cells only. This increase in nuclei size is observed in both HCT-116 and U2OS cells after etoposide treatment. Scale bar = 10 µm. **b**. Nuclear morphology changes induced by DNA damage are dose-dependent. Dose response curve measuring nuclei area in HCT-116 cells after treatment with WRNi for 24 h. Error bars represent s.d.. **c**. Validation of endogenous WRN Halo tagging in HCT-116 and U2OS cells. WRN protein levels in HCT-116$^{WRN-Halo}$ and U2OS$^{WRN-Halo}$ cells visualized by staining with JF549 dye after treatment with 10 µM of HaloTag degraders, PROTAC-E or PROTAC-3, for 24 h. Scale bar = 10 µm. **d**. Treatment with Halo-PROTAC-3 leads to proteasomal dependent degradation of WRN^{Halo}. Further validation of the WRN^{Halo} tag, showing Western blot analysis of HCT-116-WRN^{Halo} cells after treatment with 10 µM PROTAC-3 in the presence or absence of 2 nM CFZ or 5 nM MLN-4924 for 24 h. PROTAC-3 uses CUL2^{VHL} as a ligase. Therefore, CUL2 inhibition via MLN-4924 leads to a rescue of the WRN^{Halo} degradation phenotype. GAPDH was used as a loading control. **e**. Wild type HCT-116 cells and HCT-116-WRN^{Halo} cells have identical responses to WRNi, suggesting WRN^{Halo} is functional. Dose response curves of HCT-116^{WT} and HCT-116-WRN^{Halo} measuring DNA damage induction. Error bars represent s.d.. **f.** WRNi leads to a dose-dependent increase of WRN molecules bound to chromatin. Distribution of diffusive states for WRN^{Halo} with increasing concentrations of WRNi. WRNi leads to a decrease of free-disusing WRN molecules with a concomitant increase in chromatin-bound WRN molecules. **g**. WRNi does not lead to chromatin trapping in MSS cells. SMT measurements show that the distribution of WRN^{Halo} diffusive states inU2OS$^{WRN-Halo}$ cells remains unchanged in the presence or absence of 10 µM WRNi. Shaded area represent s.d.. **h** and **i**. WRN diffusion coefficient (**h**) and protein levels (**i**) remain unchanged upon DNA damage induction. Kinetic SMT of HCT-116-WRN^{Halo} after treatment with 10 µM WRNi or Etopo over the indicated time points. Error bars represent s.e.m.. CFZ is carfilzomib; DMSO is dimethylsulfoxide; WRNi is HRO761; Etopo is etoposide. MW is molecular weight.

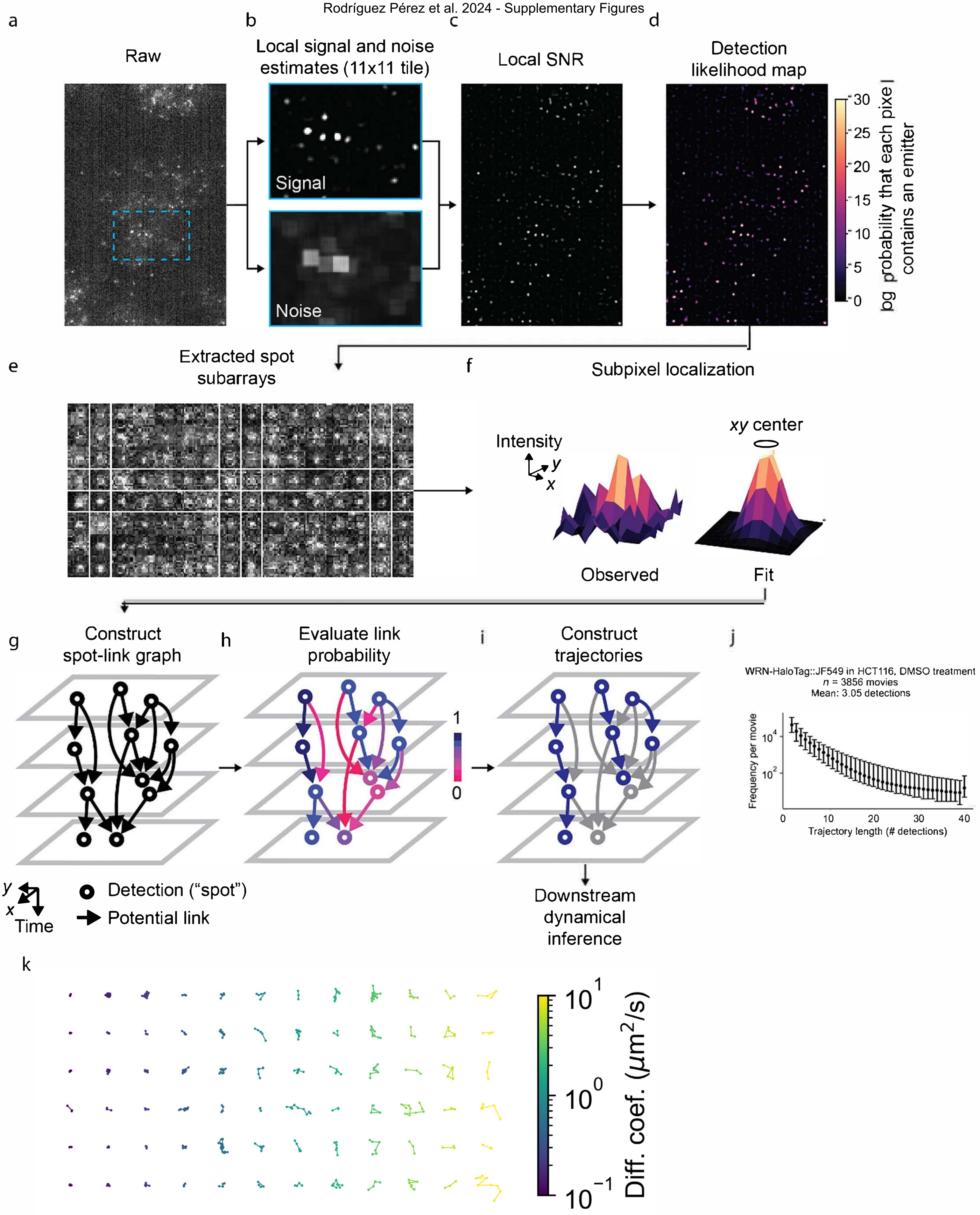
a
Raw
b
Local signal and noise estimates (11x11 tile)
Signal
Noise
c
Local SNR
d
Detection likelihood map
log probability that each pixel contains an emitter
30
25
20
15
10
5
0
Subpixel localization
e
Extracted spot subarrays
f
Intensity
y
x
xy center
Observed
Fit
g
Construct spot-link graph
h
Evaluate link probability
1
0
i
Construct trajectories
j
WRN-HaloTag::JF549 in HCT116, DMSO treatment
n = 3856 movies
Mean: 3.05 detections
Frequency per movie
Trajectory length (# detections)
0
10
20
30
40
Detection ("spot")
Potential link
Time
Downstream dynamical inference
k
Diff. coef. (μm²/s)

Supplementary Fig. 3:

Schematic of the single molecule tracking (SMT) image processing pipeline. **a.** Example of a raw frame from an SMT movie (WRN-HaloTag, HCT-116, DMSO treatment). **b.** The raw image is used to estimate the local signal and noise in 11x11 pixel neighborhoods, given a candidate point spread function. **c.** Local signal and noise estimates are used to estimate the local SNR. **d.** Local SNR is used to estimate the probability that each 11x11 pixel neighborhood contains a spot (Methods). **e.** 11x11 pixel subarrays are extracted around each spot from **d**. **f.** Spot subarrays are fit to a 2D integrated Gaussian point spread function model, yielding estimates for the subpixel location of the emitter. **g.** Emitter locations are used to construct a graph of potential paths of emitters through time. **h.** A Brownian motion model is used to estimate the probability of each link link. **i.** Trajectories are constructed by maximizing the marginal link probabilities, then subjected to downstream dynamical inference (e.g. state arrays) as appropriate for the task. **j.** Trajectory length histogram from a representative DMSO-treated well. **k**. Representative single molecule trajectories for WRN^{HALO} in $HCT\text{-}116^{WRN\text{-}Halo}$ were randomly sampled from DMSO conditions and colored by estimated diffusion coefficient. DMSO is dimethyl sulfoxide.

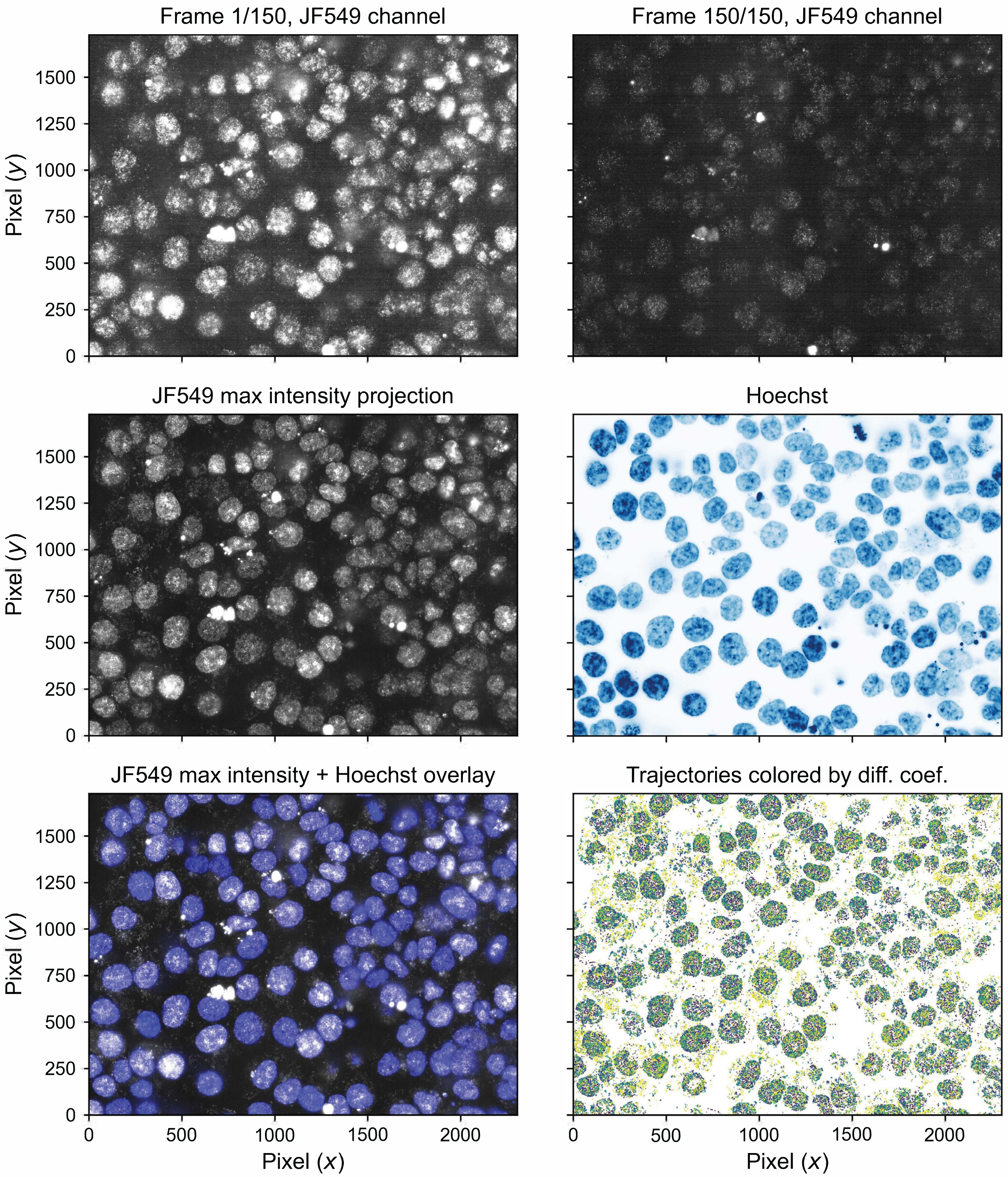
Frame 1/150, JF549 channel
Frame 150/150, JF549 channel
JF549 max intensity projection
Hoechst
JF549 max intensity + Hoechst overlay
Trajectories colored by diff. coef.
Pixel (y)
Pixel (x)
0
250
500
750
1000
1250
1500
0
500
1000
1500
2000

Supplementary Fig. 4.

Representative images of HCT-116$^{WRN\text{-}Halo}$ showing data processing steps after OLS imaging acquisition.

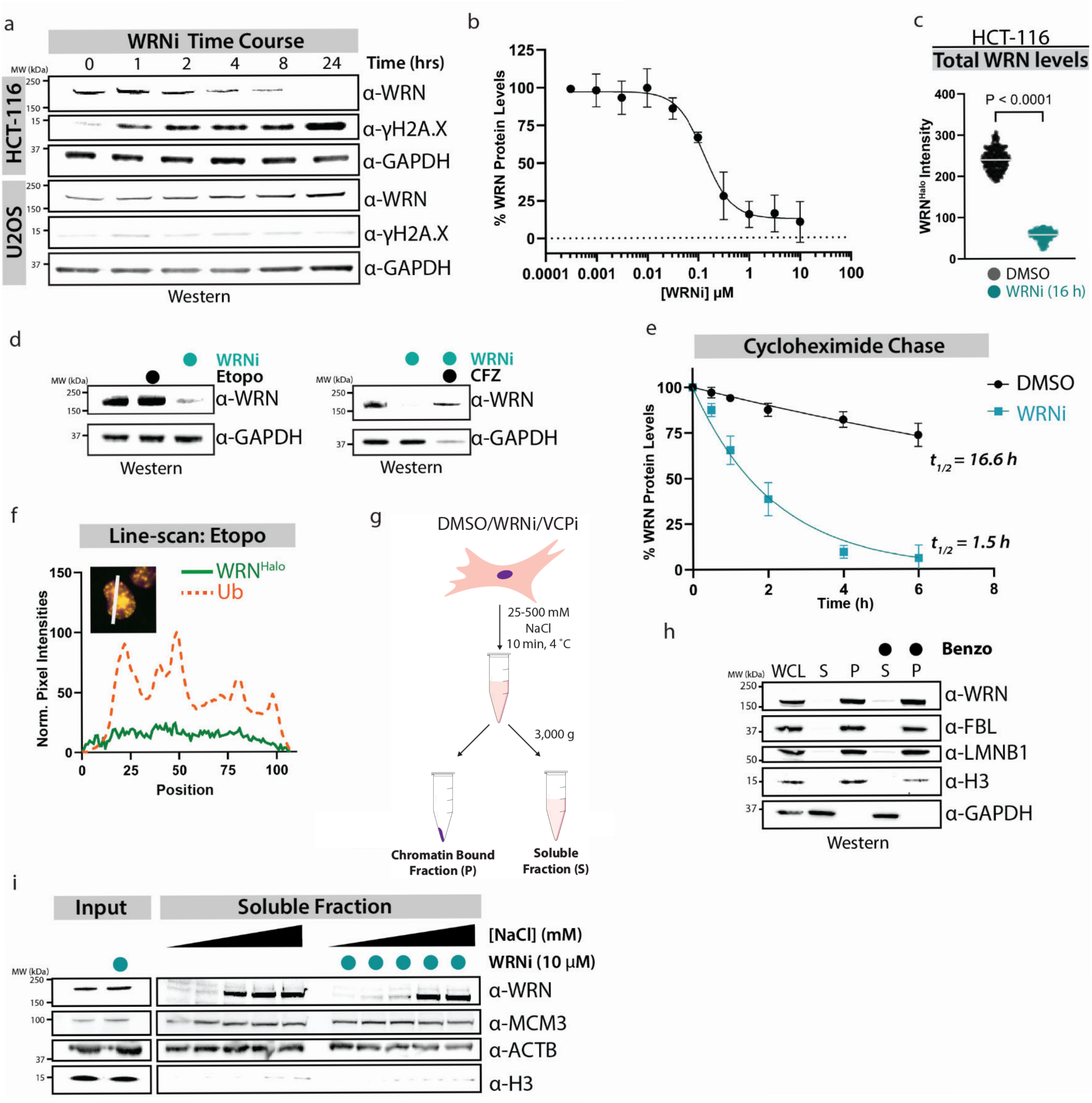
a
WRNi Time Course
0 1 2 4 8 24 Time (hrs)
HCT-116
U2OS
α-WRN
α-γH2A.X
α-GAPDH
Western
b
% WRN Protein Levels
[WRNi] μM
c
HCT-116
Total WRN levels
P < 0.0001
WRNHalo Intensity
DMSO
WRNi (16 h)
d
WRNi
Etopo
CFZ
α-WRN
α-GAPDH
Western
e
Cycloheximide Chase
DMSO
WRNi
% WRN Protein Levels
t1/2 = 16.6 h
t1/2 = 1.5 h
Time (h)
f
Line-scan: Etopo
WRNHalo
Ub
Norm. Pixel Intensities
Position
g
DMSO/WRNi/VCPi
25-500 mM NaCl
10 min, 4 °C
3,000 g
Chromatin Bound Fraction (P)
Soluble Fraction (S)
h
Benzo
WCL S P S P
α-WRN
α-FBL
α-LMNB1
α-H3
α-GAPDH
Western
i
Input
Soluble Fraction
[NaCl] (mM)
WRNi (10 μM)
α-WRN
α-MCM3
α-ACTB
α-H3
MW (kDa)

Supplementary Fig. 5:

a. Inhibition of WRN induces WRN degradation in a time-dependent manner in MSI-H cells. Steady state Western blot analysis of HCT-116 or U2OS cells treated with 10 µM WRNi over the indicated time points. GAPDH was used as a loading control. **b.** Dose response curves measuring WRN protein levels by staining HCT-116$^{WRN\text{-}Halo}$ cells with JF549 after treatment with WRNi for 24 h. Graphs represent averages from n = 3 plates, measuring 3 wells per plate and 6 FOVs per well. The curve fit was done by fitting a 4-parameter logarithmic regression curve **c.** Quantification of **Fig. 3c.** Data represents averages from *n* = 3 plates, with each individual point representing one well. **d.** Degradation of WRN is induced by WRNi, but not by general DNA damage. Western blot analysis of HCT-116 cells treated with 10 µM etoposide or WRNi. Degradation is rescued by the addition of CFZ. GAPDH was used as a loading control. **e.** Quantification of **Fig. 3b.** WRN inhibition leads to a decrease in the half-life of WRN protein. Cycloheximide (CHX) chase experiments in HCT-116 cells in the presence or absence of 10 µM WRNi show a dramatic decrease in the half-life of WRN protein upon its inhibition. Graphs represent *n* = 2 replicates. The curve fit was done by fitting a half-life decay regression curve. **f.** Line-scan quantification of etoposide treated cells from **Fig. 3d**,showing a lack of co-localization of the ubiquitin signal and the WRN signal. **g.** Schematic representation of the chromatin fractionation assay. **h**. WRN is tightly bound to chromatin under steady state conditions. Western blot analysis of subcellular fractions of HCT-116 cells fractionated in 150 mM NaCl CSK buffer. GAPDH and H3 were used as processing controls. **i.** Western blot analysis of HCT-116 inputs and soluble fractions from **Fig. 3g.** ACTB and H3 were used as processing controls. DMSO is dimethyl sulfoxide; CFZ is carfilzomib; p97i is CB-5083; WRNi is HRO761; Benzo is benzonase nuclease. P-values were calculated using a two-tailed, unpaired Student's t-test. MW is molecular weight.

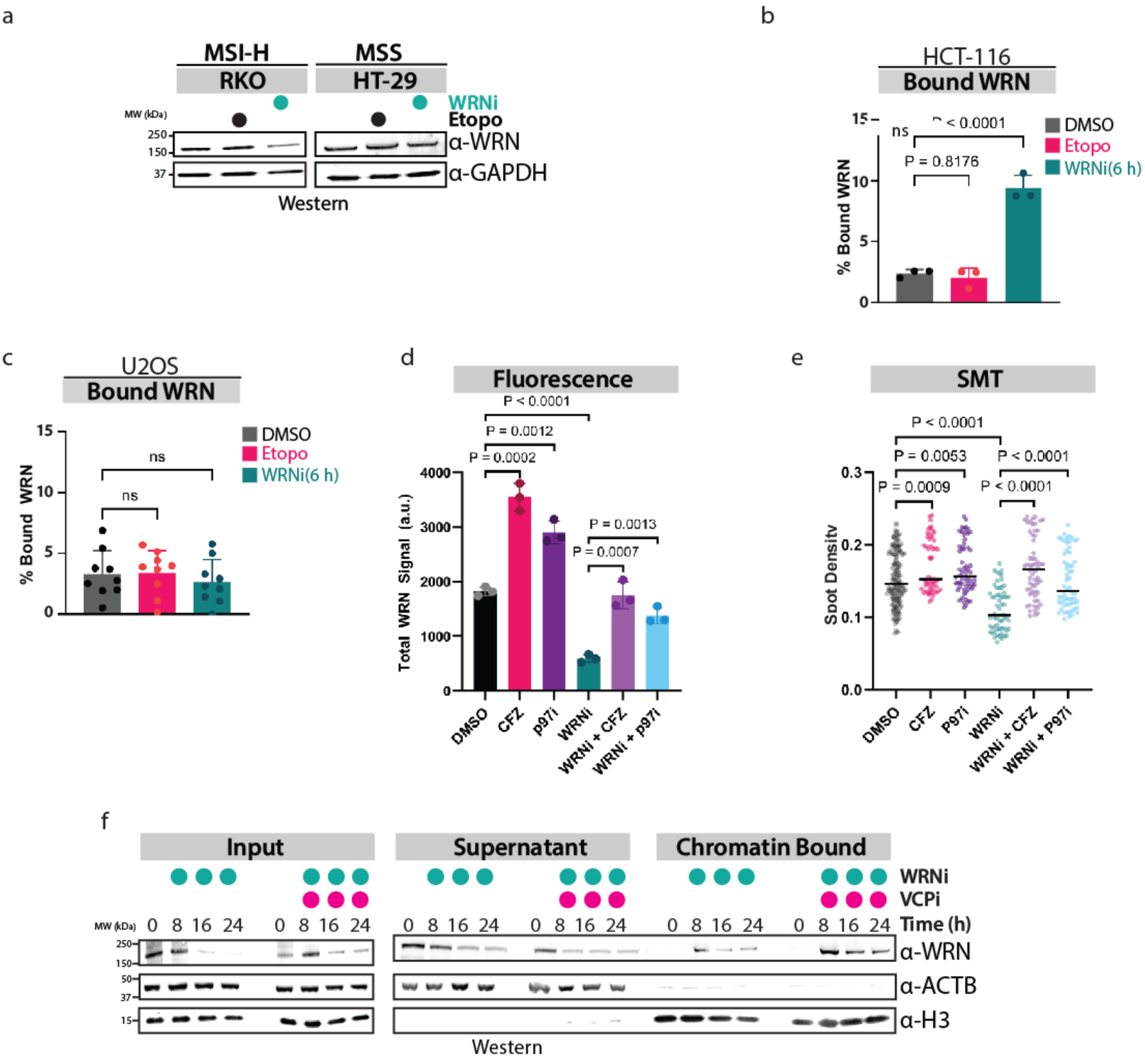
a
MSI-H
RKO
MSS
HT-29
WRNi
Etopo
MW (kDa)
250
150
37
α-WRN
α-GAPDH
Western
b
HCT-116
Bound WRN
ns
< 0.0001
P = 0.8176
DMSO
Etopo
WRNi(6 h)
% Bound WRN
15
10
5
0
c
U2OS
Bound WRN
ns
ns
DMSO
Etopo
WRNi(6 h)
% Bound WRN
15
10
5
0
d
Fluorescence
P < 0.0001
P = 0.0012
P = 0.0002
P = 0.0013
P = 0.0007
Total WRN Signal (a.u.)
4000
3000
2000
1000
0
DMSO
CFZ
p97i
WRNi
WRNi + CFZ
WRNi + p97i
e
SMT
P < 0.0001
P = 0.0053
P < 0.0001
P = 0.0009
P < 0.0001
Spot Density
0.3
0.2
0.1
0.0
DMSO
CFZ
P97i
WRNi
WRNi + CFZ
WRNi + P97i
f
Input
Supernatant
Chromatin Bound
WRNi
VCPi
Time (h)
MW (kDa)
0 8 16 24 0 8 16 24
250
150
50
37
15
α-WRN
α-ACTB
α-H3
Western

Supplementary Fig. 6:

a. WRN degradation upon its inhibition is MSI-H dependent. The indicated MSI-H or MSS cell lines were treated as indicated and analyzed by Western blot with the indicated antibodies. GAPDH was used as loading controls **b.** Quantification of trapped WRN in **Fig. 3d**. Bar graphs represent sample means, each point represents well-level average from *n = 3* plates. **c**. Quantification of trapped WRN in **Fig 3j.** **d.** Quantifications of **Fig. 3k**. Bar graphs are the mean of *n = 3* plates, each point represents the well-level average. **e**. SMT can be used to measure protein degradation. SMT was used to measure WRN molecules after inhibition of the p97/VCP-proteasome pathway, showing a rescue in protein degradation. Each point represents the average WRN spot density within all the nuclei in an FOV. n= 4 plates. Lines represent sample medians. **f**. Chromatin trapping of WRN is p97/VCP dependent. Western blot analysis of HCT-116 cells treated with WRNi in the presence or absence of p97/VCPi over the indicated time points. All error bars represent s.d.. ACTB and H3 were used as processing controls. DMSO is dimethyl sulfoxide, WRNi is HRO761; Etopo is etoposide; CFZ is carfilzomib; p97i is CB-5083; E1i is TAK-243; SUMOi is ML-792.P-values were calculated using a two-tailed, unpaired Student's t-test. ns = not significant.

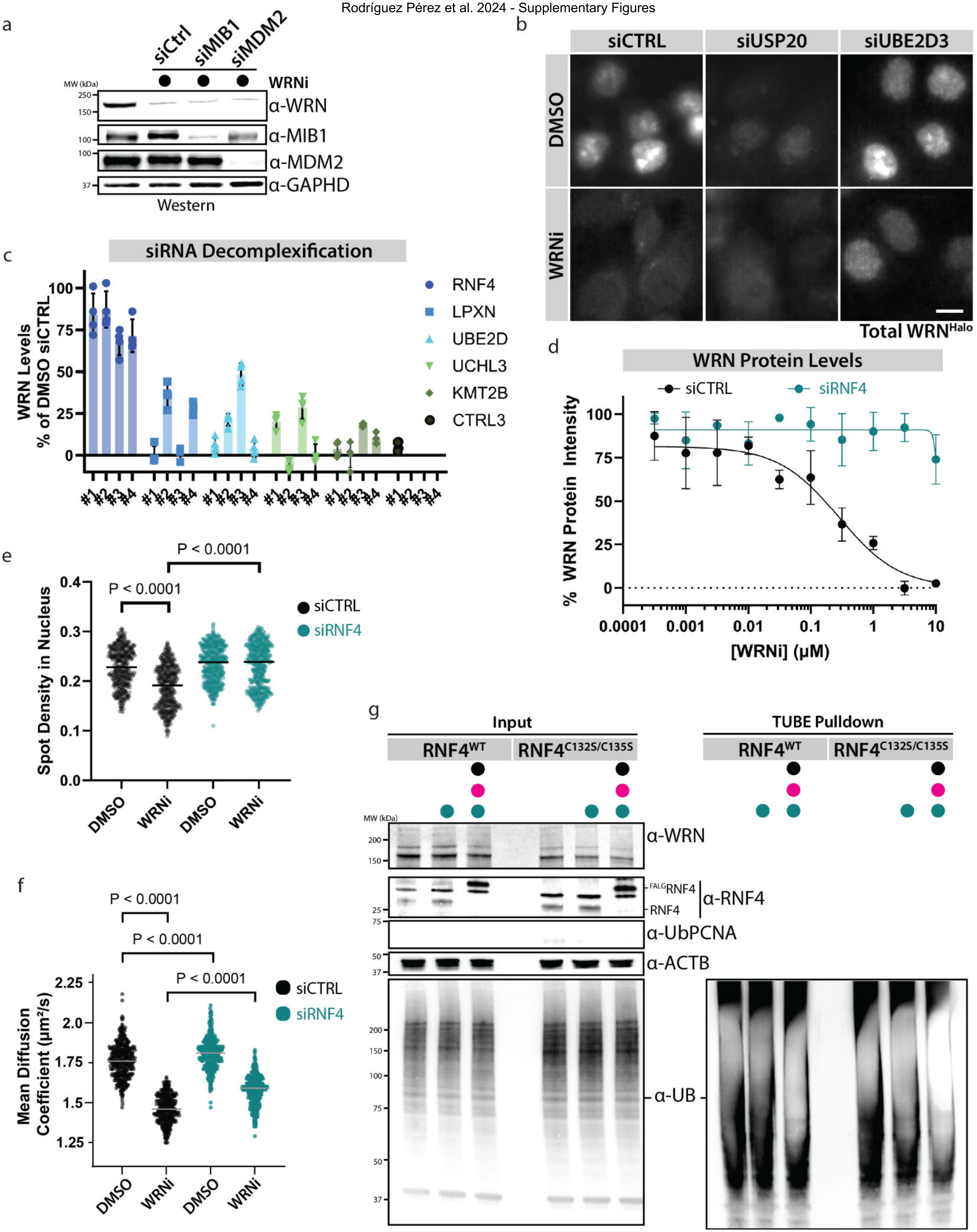
a
siCtrl
siMIB1
siMDM2
WRNi
MW (kDa)
250
150
100
100
37
α-WRN
α-MIB1
α-MDM2
α-GAPHD
Western
b
siCTRL
siUSP20
siUBE2D3
DMSO
WRNi
Total WRN^Halo
c
siRNA Decomplexification
WRN Levels
% of DMSO siCTRL
100
75
50
25
0
RNF4
LPXN
UBE2D
UCHL3
KMT2B
CTRL3
#1 #2 #3 #4
d
WRN Protein Levels
siCTRL
siRNF4
% WRN Protein Intensity
100
75
50
25
0
0.0001
0.001
0.01
0.1
1
10
[WRNi] (μM)
e
P < 0.0001
P < 0.0001
Spot Density in Nucleus
0.4
0.3
0.2
0.1
0.0
siCTRL
siRNF4
DMSO
WRNi
DMSO
WRNi
f
P < 0.0001
P < 0.0001
P < 0.0001
Mean Diffusion Coefficient (μm²/s)
2.25
2.0
1.75
1.5
1.25
siCTRL
siRNF4
DMSO
WRNi
DMSO
WRNi
g
Input
TUBE Pulldown
RNF4^WT
RNF4^C132S/C135S
RNF4^WT
RNF4^C132S/C135S
MW (kDa)
200
150
25
75
50
37
200
150
100
75
50
37
α-WRN
FALGRNF4
RNF4
α-RNF4
α-UbPCNA
α-ACTB
α-UB

Supplementary Fig. 7:

a. Previously reported E3 ligases of WRN are not responsible for the WRNi-dependent degradation phenotype. HCT-116 were treated with indicated siRNA oligos for 24 h, and subsequently treated with 10 µM WRNi for 16 h and analyzed by Western, showing that depletion of the indicated E3 ligases does not rescue the WRN degradation phenotype. GAPDH was used as processing controls. **b.** Identification of additional ubiquitin pathway regulators of WRN regulation. Representative images of HCT-116 cells after depletion of the indicated ubiquitin pathway regulators. Scale bar = 10 µm. **c.** Quantification of decomplexified siRNA screen hits from Fig. 4c after treatment of HCT-116$^{WRN\text{-}Halo}$ with indicated siRNA oligos for 24 h and then subsequent treatment with 10 µM WRNi for 24 h. Bar graphs are the averages from quantification of WRN protein levels for each indicated siRNA oligo with *n = 3* plates. **d**. RNF4 depletion rescues the WRN degradation phenotype. Dose response curves measuring WRN protein levels by imaging HCT-116$^{WRN\text{-}Halo}$ cells treated with the indicated siRNAs for 24 h, and subsequent treatment with WRNi for 24 h. Graphs represent averages from n = 3 plates, measuring 3 wells per plate and 6 FOVs per well. **e**. SMT can be used to quantify protein degradation. Depletion of RNF4 rescues WRN protein levels after WRN inhibition. WRNi dot plots showing the WRN nuclear spot density from SMT experiments after co-treatment with siRNF4 and either DMSO or WRNi. Each point represents the average WRN spot density within all the nuclei in an FOV. *n = 4* plates. Lines represent sample medians. **f**. RNF4 depletion leads to a slight but significant increase in the mean diffusion coefficient of WRN. Dot plots of WRN diffusion coefficient via SMT after co-treatment with siRNF4 and either DMSO or WRNi. Each point represents the average WRN diffusion coefficient within all the nuclei in an FOV. *n = 4* plates. Lines represent sample medians. **g**. Inputs and total ubiquitin loading controls for TUBE PD from **Fig. 4j**. ACTB was used as loading controls; UB was used as a processing control. WRNi is HRO761. MW is molecular weight. All error bars represent s.d

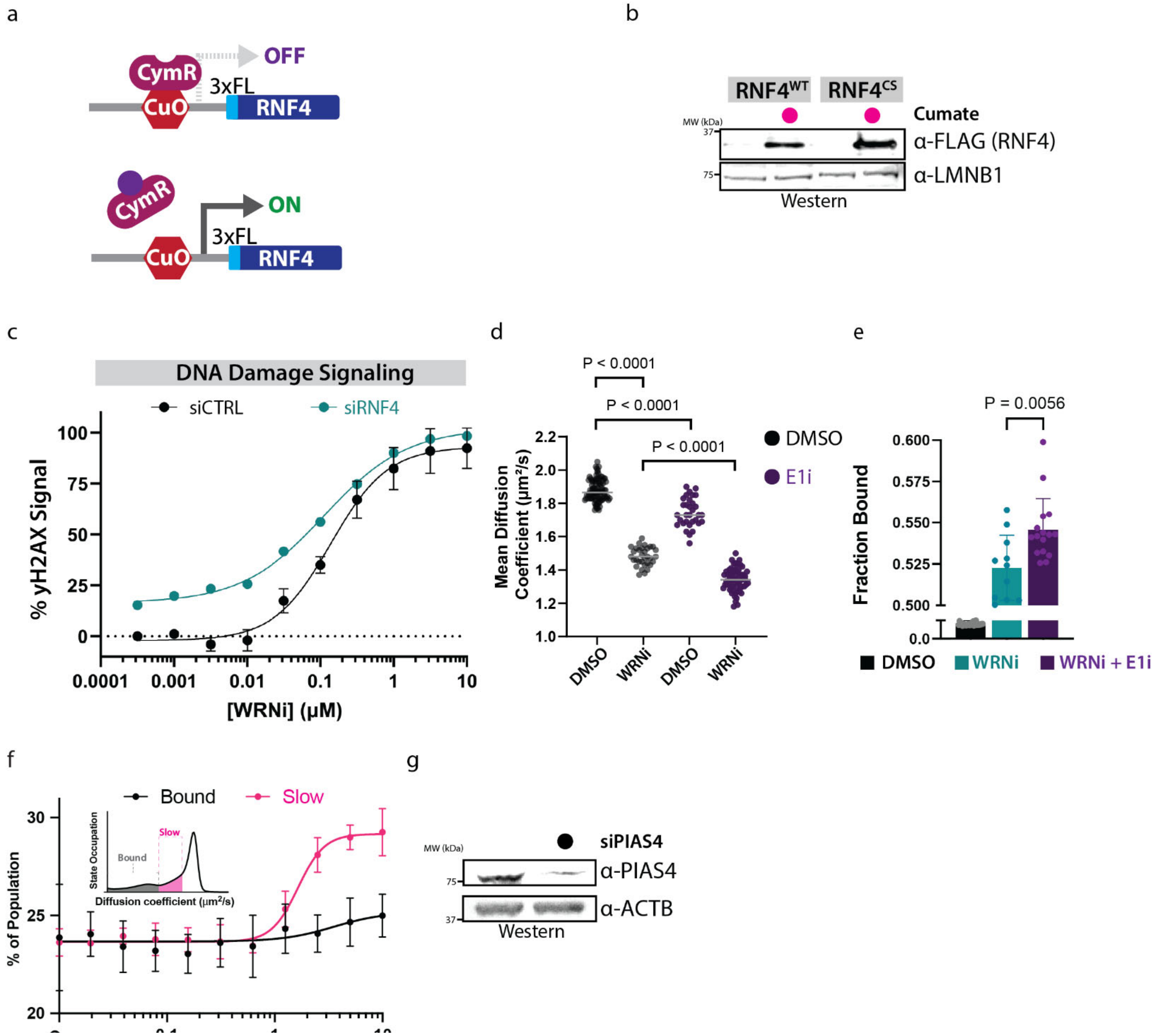
a
CymR
OFF
3xFL
CuO
RNF4
CymR
ON
3xFL
CuO
RNF4
b
RNF4WT
RNF4CS
Cumate
MW (kDa)
37
75
α-FLAG (RNF4)
α-LMNB1
Western
c
DNA Damage Signaling
siCTRL
siRNF4
% γH2AX Signal
100
75
50
25
0
0.0001
0.001
0.01
0.1
1
10
[WRNi] (μM)
d
P < 0.0001
P < 0.0001
P < 0.0001
DMSO
E1i
Mean Diffusion Coefficient (μm²/s)
2.2
2.0
1.8
1.6
1.4
1.2
1.0
DMSO
WRNi
DMSO
WRNi
e
P = 0.0056
Fraction Bound
0.600
0.575
0.550
0.525
0.500
0.0
DMSO
WRNi
WRNi + E1i
f
Bound
Slow
% of Population
30
25
20
State Occupation
Bound
Slow
Diffusion coefficient (μm²/s)
1
g
siPIAS4
MW (kDa)
75
37
α-PIAS4
α-ACTB
Western

Supplementary Fig. 8:

a. Schematic of the cumate inducible system used for functional validation of the WRN degradation phenotype. **b.** Stable gene induction is cumate dependent. Western blot analysis showing the expression of the indicated constructs of RNF4 after cumate treatment. LMNB was used as a loading control. **c.** Depletion of RNF4 exacerbates DNA damage induced by WRNi. HCT-116 cells were treated with the indicated siRNAs for 24 h, and subsequently subjected to a dose response of WRNi for 16 h. DNA damage was assessed by measuring γH2A.X staining. Graphs represent averages from *n* = 3 plates, measuring 3 wells per plate and 6 FOVs per well. All curve fits were done by fitting a 4-parameter logarithmic regression curve**. d.** The ubiquitin pathway is involved in regulating WRN dynamics. Dot plots of WRN diffusion coefficient via SMT after co-treatment with the ubiquitin-activating enzyme (E1) inhibitor (E1i) and either DMSO or WRNi. E1i treatment caused a reduction in the diffusion coefficient of WRN. This decrease in diffusion was exacerbated by cotreatment with WRNi. Each point represents the average WRN diffusion coefficient within all the nuclei in an FOV. n = 4 plates. **e**. Treatment with E1i shows a dose-dependent increase in the slow fraction of WRN, suggesting that the ubiquitin pathway regulates WRN dynamics. Dose response curves with E1i measuring the “bound” and “slow” fractions of WRN protein. The inset indicates how diffusive states are classified. **f**. Bar graph quantification of the fraction bound of WRN^{Halo} protein after WRNi treatment in the presence or absence of E1i. Each dot represent a well, *n* = 2 plates. **g.** Western blot analysis of HCT-116 cells showing the depletion of PIAS4 following siPIAS4 oligo treatments. ACTB was used as loading control. DMSO is dimethyl sulfoxide;WRNi is HRO761; E1i is TAK-243. MW is molecular weight. All error bars represent s.d..

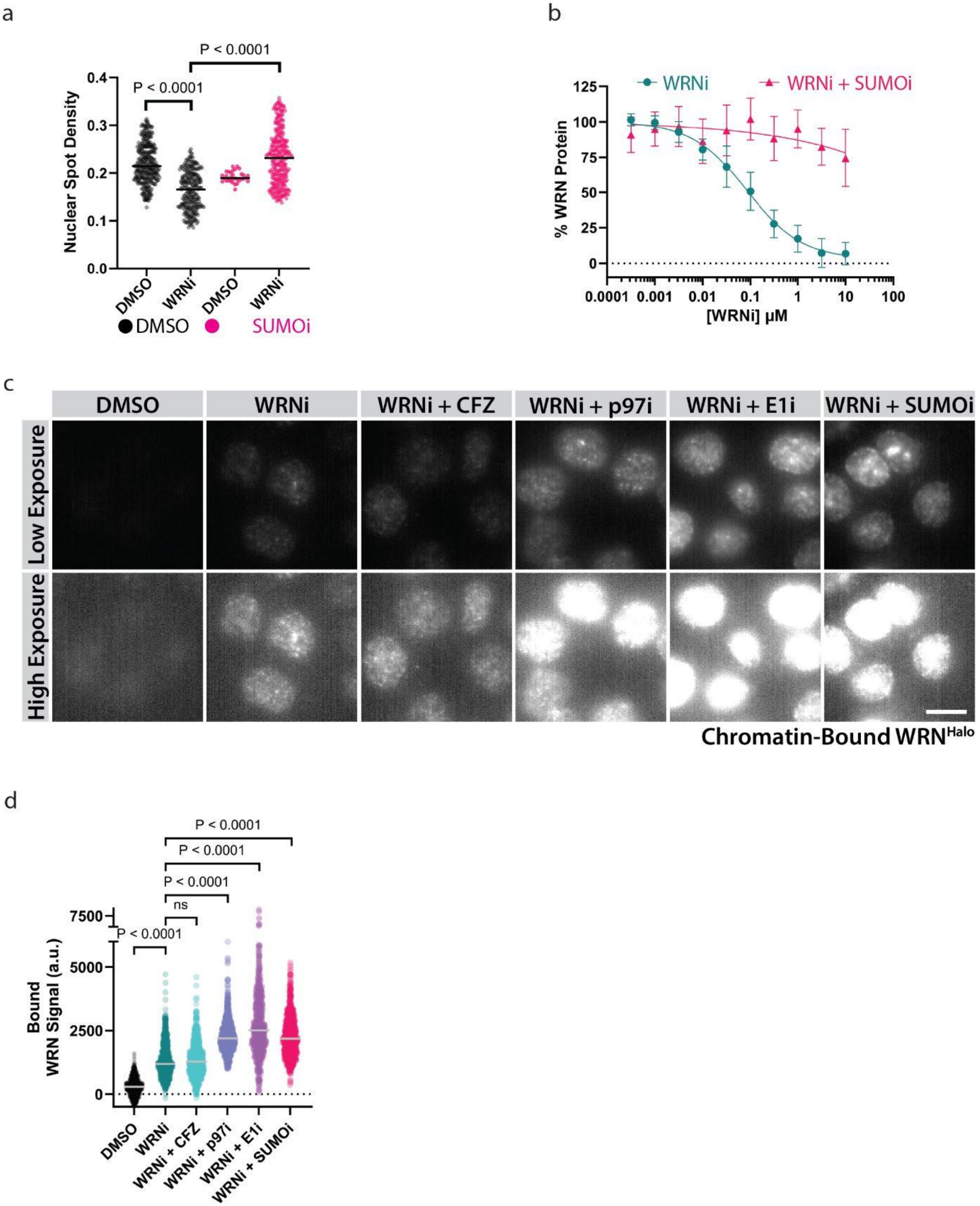
a
P < 0.0001
P < 0.0001
Nuclear Spot Density
0.4
0.3
0.2
0.1
0.0
DMSO
WRNi
DMSO
WRNi
DMSO
SUMOi
b
WRNi
WRNi + SUMOi
% WRN Protein
125
100
75
50
25
0
0.0001
0.001
0.01
0.1
1
10
100
[WRNi] μM
c
DMSO
WRNi
WRNi + CFZ
WRNi + p97i
WRNi + E1i
WRNi + SUMOi
Low Exposure
High Exposure
Chromatin-Bound WRN^Halo
d
P < 0.0001
P < 0.0001
P < 0.0001
ns
P < 0.0001
Bound WRN Signal (a.u.)
7500
5000
2500
0
DMSO
WRNi
WRNi + CFZ
WRNi + p97i
WRNi + E1i
WRNi + SUMOi

Supplementary Fig. 9:

a. SMT can be used to elucidate molecular regulatory pathways, such as protein degradation. Inhibition of SUMOylation rescues WRN protein levels after WRN inhibition. WRNi dot plots showing the WRN nuclear spot density from SMT experiments after co-treatment with SUMOi and either DMSO or WRNi. Each point represents the average spot density within all the nuclei in an FOV. n = 4 plates. Lines represent sample medians. **b**. Inhibition of SUMOylation prevents WRN degradation by WRNi. Dose response curves measuring WRN protein levels in HCT-116$^{WRN\text{-}Halo}$ cells treated with WRNi in the presence or absence of 1 μM SUMOi for 24 h. Graphs represent averages from *n* = 3 plates, measuring 3 well per plate and 6 FOVs per well. **c.** The SUMO-Ubiquitin-p97/VCP axis is required to remove trapped WRN from chromatin. Treatment of HCT-116-WRN^{Halo} cells with 10 μM WRNi in the presence or absence of 1 μM of CFZ, p97i, E1i, or SUMOi, followed by detergent extraction and imaging. **d**. Dot plot quantification of **c**. Each point represents an individual cell, measuring the nuclear intensity of WRN. Bars represent the means of the populations. DMSO is dimethyl sulfoxide, WRNi is HRO761; CFZ is carfilzomib; p97i is CB-5083; E1i is TAK-243; SUMOi is ML-792. P-values were calculated using a two-tailed, unpaired Student’s t-test. ns = not significant.

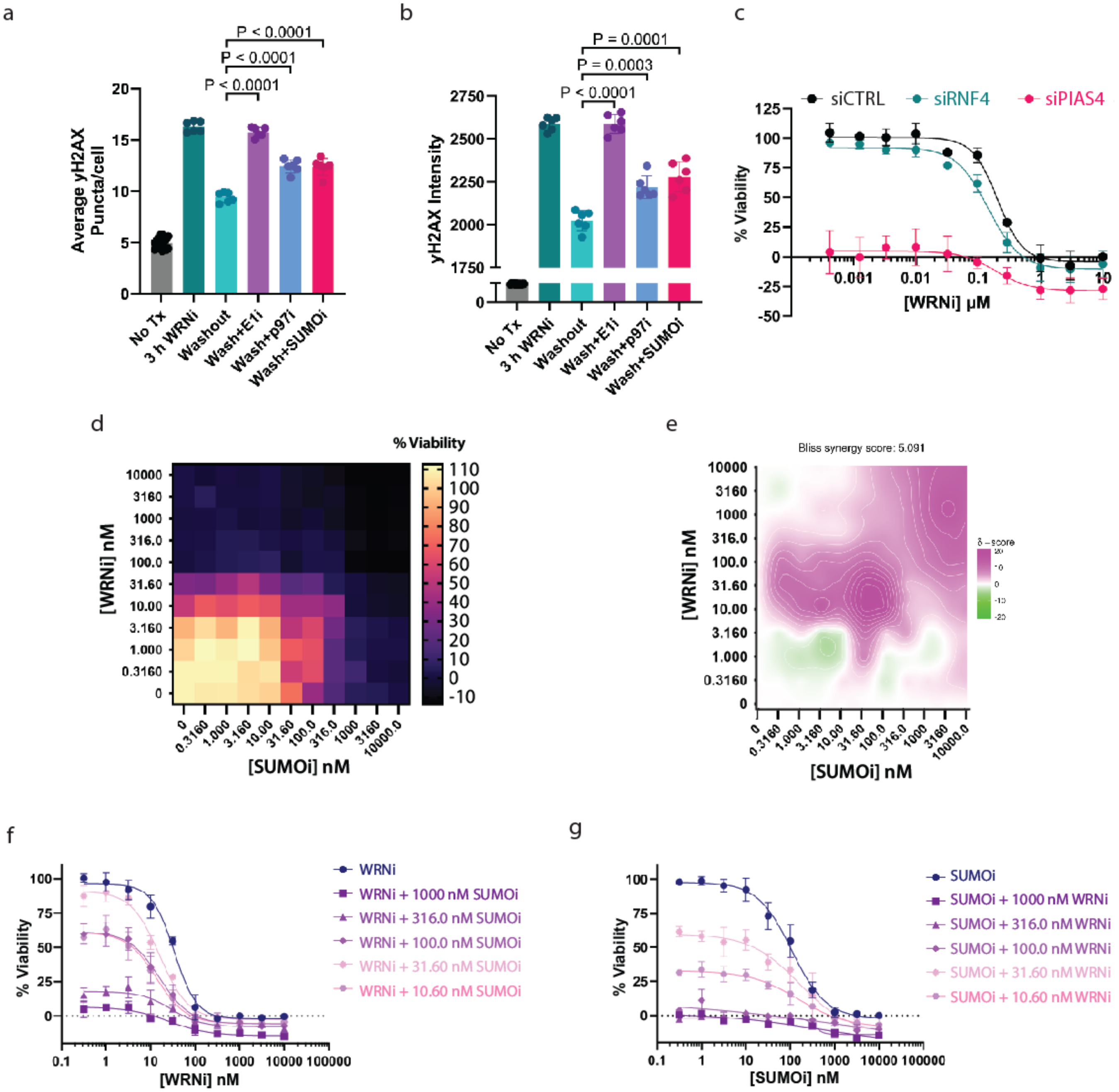

h

Drug combination	Synergy score	Most synergistic area score	Method
SUMOi - WRNi	5.09	12.96	Bliss

Chosen parameters:

Readout: viability ; Baseline correction: Yes

Supplementary Fig. 10:

a and **b**. Quantification of the washout experiment in **Fig. 5l**. **a** quantifies the average number of γH2A.X puncta per nuclei in an FOV, and **b** quantifies the average γH2A.X nuclear intensities of an FOV. Bar graphs in both **a** and **b** represent the average of n = 3 plates. Each data point represents the average of one well, containing 6 FOVs. **c**. WRNi compound efficacy is independent of WRN degradation but shows potential sensitization to inhibition of the PIAS4-RNF4 axis. HCT-116 cells were treated with the indicated siRNAs for 24 h, followed by WRNi treatment at the indicated doses for 48 h. Cell viability was measured using a CTG2 kit. Graphs represent averages from n = 3 plates. **d**. Co-treatment of WRNi with SUMOi has potential synergy. Dose response matrix of both WRNi and SUMOi with the indicated concentrations of compound. HCT-116 cells were treated with the indicated dose combinations for 48 h. Viability was measured via CTG2. **e**. Synergy map of **d**, showing Bliss synergy scores for SUMOi and WRNi. The average Bliss synergy score is 5.091, with the most synergistic area score being 12.96, suggesting some potential synergy. **f** and **g**. Quantifications of **d**, showing dose response plots for the indicated concentration combinations. Graphs represent averages from n = 3 plates. **h**. SynergyFinder summary table. All curve fits were done by fitting a 4-parameter logarithmic regression curve. DMSO is dimethyl sulfoxide; WRNi is HRO761; E1 is TAK-243; SUMOi is ML-792; p97i is CB-5083. P-values were calculated using a two-tailed, unpaired Student’s t-test. All error bars represent s.d..

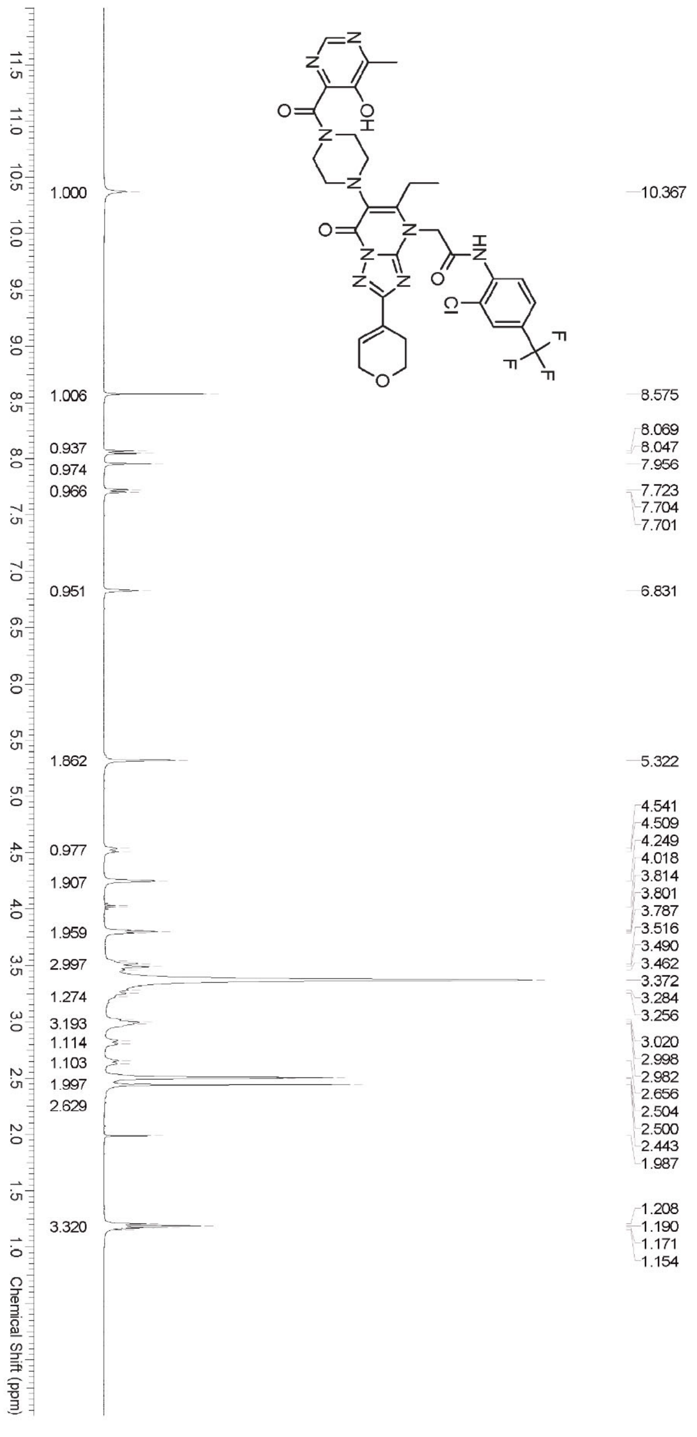

Supplementary Fig. 11:

^{1}H NMR spectrum was recorded on a Bruker ADVANCE spectrometer at 400 MHz. The chemical shifts are given in parts per million (ppm) on a delta (δ) scale. The solvent peak DMSO-d_6 = 2.50 ppm was used as a reference value for ^{1}H NMR.

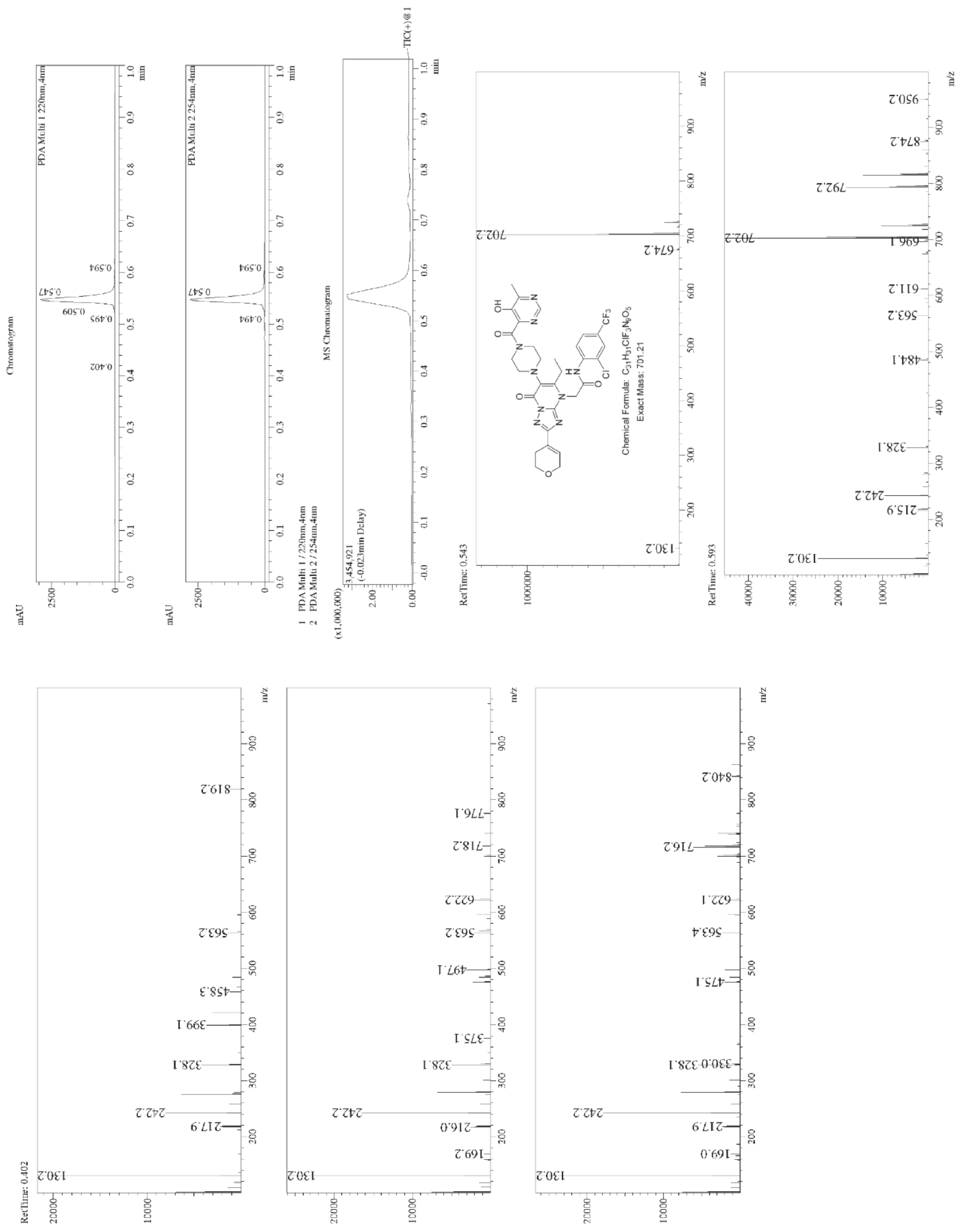

Supplementary Fig. 12:

Molecular weight of Compound 1 was confirmed by liquid chromatography mass spectrometry (ESI+) calcd for $C_{31}H_{31}ClF_3N_9O_5$ [M + H]+ 702.094, found 702.2.

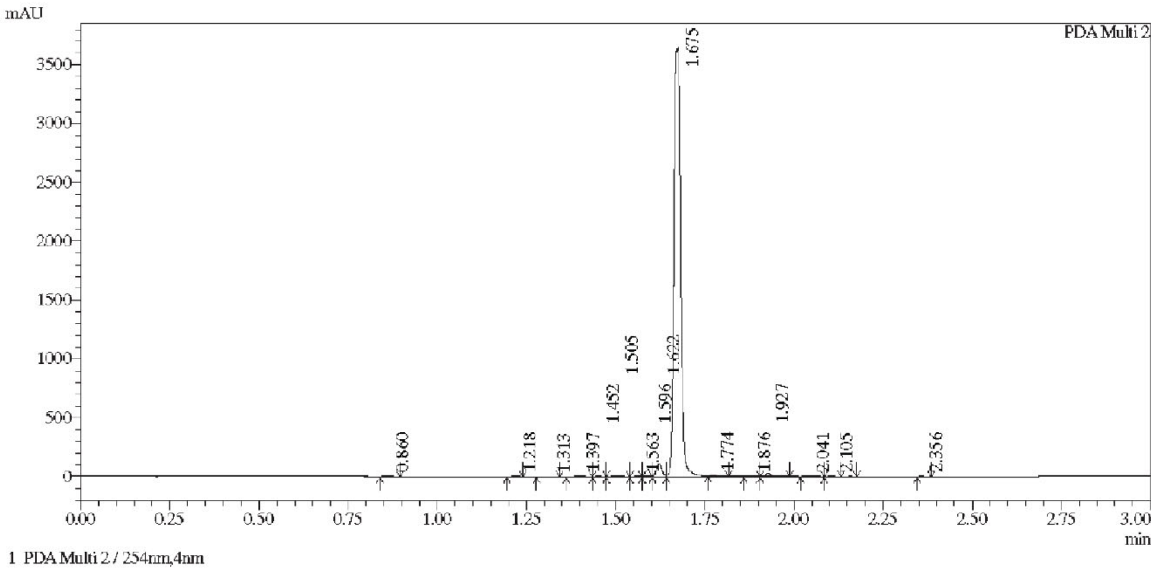

Supplementary Fig. 13:

Purity of Compound 1 was determined to be >95% by high performance liquid chromatography.
