# Supplementary material for: WRN inhibition leads to its chromatin-associated degradation via the PIAS4-RNF4-p97/VCP axis
Source: Nat Commun. 2024 Jul 18;15:6059. doi: 10.1038/s41467-024-50178-3 (PMC11258360; doi:10.1038/s41467-024-50178-3)
Supplement: Supplementary file 3 — Description of Supplementary Information [file 41467_2024_50178_MOESM3_ESM.pdf]

Description of Additional Supplementary Files

File Name: Supplementary Movie 1
Description: Raw movies of HCT-116WRN-Halo treated with WRNi or DMSO. Single molecule data processing is performed on images to extract diffusion coefficients and diffusive states distributions.
