# Supplementary material for: WRN inhibition leads to its chromatin-associated degradation via the PIAS4-RNF4-p97/VCP axis
Source: Nat Commun. 2024 Jul 18;15:6059. doi: 10.1038/s41467-024-50178-3 (PMC11258360; doi:10.1038/s41467-024-50178-3)
Supplement: Supplementary file 5 — Reporting Summary [file 41467_2024_50178_MOESM5_ESM.pdf]

nature portfolio

Corresponding author(s): Fernando Rodréguez Pérez
Stephen Basham

Last updated by author(s): Jun 24, 2024

Reporting Summary

Nature Portfolio wishes to improve the reproducibility of the work that we publish. This form provides structure for consistency and transparency in reporting. For further information on Nature Portfolio policies, see our Editorial Policies and the Editorial Policy Checklist.

Statistics

For all statistical analyses, confirm that the following items are present in the figure legend, table legend, main text, or Methods section.

n/a	Confirmed	
☐	☒	The exact sample size (*n*) for each experimental group/condition, given as a discrete number and unit of measurement
☐	☒	A statement on whether measurements were taken from distinct samples or whether the same sample was measured repeatedly
☐	☒	The statistical test(s) used AND whether they are one- or two-sided<br>*Only common tests should be described solely by name; describe more complex techniques in the Methods section.*
☒	☐	A description of all covariates tested
☒	☐	A description of any assumptions or corrections, such as tests of normality and adjustment for multiple comparisons
☐	☒	A full description of the statistical parameters including central tendency (e.g. means) or other basic estimates (e.g. regression coefficient) AND variation (e.g. standard deviation) or associated estimates of uncertainty (e.g. confidence intervals)
☒	☐	For null hypothesis testing, the test statistic (e.g. *F*, *t*, *r*) with confidence intervals, effect sizes, degrees of freedom and *P* value noted<br>*Give P values as exact values whenever suitable.*
☒	☐	For Bayesian analysis, information on the choice of priors and Markov chain Monte Carlo settings
☒	☐	For hierarchical and complex designs, identification of the appropriate level for tests and full reporting of outcomes
☒	☐	Estimates of effect sizes (e.g. Cohen's *d*, Pearson's *r*), indicating how they were calculated

Our web collection on statistics for biologists contains articles on many of the points above.

Software and code

Policy information about availability of computer code

Data collection

For Confocal Microscopy Data collection, data was acquired using a Molecular Devices IXM6 Confocal Miscroscope.

For SMT Data: Eikon supports allowing researchers to access the data within this manuscript and wants this data to be a resource for the research community. The process of generating single molecule tracking data as implemented in this manuscript is both data storage and data processing intensive. The data in this manuscript alone is 100s of terabytes, and there is no database in existence appropriate to host or make available these data. Similarly, while the core of the image processing and tracking algorithms are implementations of the works we cite in our methods section, the specific software written for the purposes of processing and analyzing these large image sets was developed with the specific compute and storage infrastructure of Eikon in mind. Even with significant effort from the authors to remove any proprietary information from the codebase, it would be unreasonable to expect a reader to be able to install and successfully run any one piece of the processing pipeline.

It is hard to anticipate all the different ways someone might want to examine these datasets. Rather than trying to anticipate which formats will be useful to future researchers, we hope that interested parties work with us to identify the data they are interested in and in what format to be useful to their work. Other materials and reagents in this manuscript will be made available via a Materials Transfer Agreement with the requesting party for research use.

Data analysis

For Confocal Microscopy Data analysis, data was processed using a Molecular Devices custom module editor version 6.1

For SMT data analysis, Eikon supports allowing researchers to access the data within this manuscript and wants this data to be a resource for the research community. The process of generating single molecule tracking data as implemented in this manuscript is both data storage and

data processing intensive. The data in this manuscript alone is 100s of terabytes, and there is no database in existence appropriate to host or make available these data. Similarly, while the core of the image processing and tracking algorithms are implementations of the works we cite in our methods section, the specific software written for the purposes of processing and analyzing these large image sets was developed with the specific compute and storage infrastructure of Eikon in mind. Even with significant effort from the authors to remove any proprietary information from the codebase, it would be unreasonable to expect a reader to be able to install and successfully run any one piece of the processing pipeline.

It is hard to anticipate all the different ways someone might want to examine these datasets. Rather than trying to anticipate which formats will be useful to future researchers, we hope that interested parties work with us to identify the data they are interested in and in what format to be useful to their work. Other materials and reagents in this manuscript will be made available via a Materials Transfer Agreement with the requesting party for research use.

For manuscripts utilizing custom algorithms or software that are central to the research but not yet described in published literature, software must be made available to editors and reviewers. We strongly encourage code deposition in a community repository (e.g. GitHub). See the Nature Portfolio guidelines for submitting code & software for further information.

Data

Policy information about availability of data

All manuscripts must include a data availability statement. This statement should provide the following information, where applicable:

- Accession codes, unique identifiers, or web links for publicly available datasets
- A description of any restrictions on data availability
- For clinical datasets or third party data, please ensure that the statement adheres to our policy

All non-SMT data pertinent to the findings presented in this article can be found within the article, supplementary figures, and source data.

Processed SMT data can be found within the source data file. However, there is no appropriate database in existence at present in order to deposit our raw Single Molecule Tracking (SMT) data, and similarly the compute power necessary to process the results is not accessible to most. Parties interested in accessing the data contained within the manuscript should contact the corresponding author to determine which non-proprietary data are needed and in what format they will be provided though a Data Use Agreement with Eikon Therapeutics. Other materials will be made available through a Materials Transfer Agreement with Eikon Therapeutics by contacting the corresponding author.

Research involving human participants, their data, or biological material

Policy information about studies with human participants or human data. See also policy information about sex, gender (identity/presentation), and sexual orientation and race, ethnicity and racism.

Reporting on sex and gender	N/A
Reporting on race, ethnicity, or other socially relevant groupings	N/A
Population characteristics	N/A
Recruitment	N/A
Ethics oversight	N/A

Note that full information on the approval of the study protocol must also be provided in the manuscript.

Field-specific reporting

Please select the one below that is the best fit for your research. If you are not sure, read the appropriate sections before making your selection.

[x] Life sciences [ ] Behavioural & social sciences [ ] Ecological, evolutionary & environmental sciences

For a reference copy of the document with all sections, see nature.com/documents/nr-reporting-summary-flat.pdf

Life sciences study design

All studies must disclose on these points even when the disclosure is negative.

Sample size	Sample sizes are described in the figure legends of the manuscript
Data exclusions	N/A
Replication	Replication is described in the figure legends of the manuscript
Randomization	N/A

Blinding	N/A

Reporting for specific materials, systems and methods

We require information from authors about some types of materials, experimental systems and methods used in many studies. Here, indicate whether each material, system or method listed is relevant to your study. If you are not sure if a list item applies to your research, read the appropriate section before selecting a response.

Materials & experimental systems

n/a	Involved in the study	
☐	☒	Antibodies
☐	☒	Eukaryotic cell lines
☒	☐	Palaeontology and archaeology
☐	☒	Animals and other organisms
☒	☐	Clinical data
☒	☐	Dual use research of concern
☒	☐	Plants

Methods

n/a	Involved in the study	
☒	☐	ChIP-seq
☒	☐	Flow cytometry
☒	☐	MRI-based neuroimaging

Antibodies

Antibodies used	All antibodies use are described in the methods of the manuscript
Validation	Validation of antibody reagents is described in the methods of the manuscript or from vendor/previously published information

Eukaryotic cell lines

Policy information about cell lines and Sex and Gender in Research

Cell line source(s)	ATCC
Authentication	Engineered cell lines were validated by Sanger sequencing. Given that all cell lines used in this study were obtained from ATCC, no authentication was performed.
Mycoplasma contamination	All cell lines are tested for Mycoplasma upon receiving as per Eikon Therapeutics QC standards. All cell line are negative.
Commonly misidentified lines (See ICLAC register)	N/A

Animals and other research organisms

Policy information about studies involving animals; ARRIVE guidelines recommended for reporting animal research, and Sex and Gender in Research

Laboratory animals	8 week old female Crl:NU(NCr)-Foxn1nu outbred mice (Charles River, strain code: 490)
Wild animals	N/A
Reporting on sex	N/A
Field-collected samples	N/A
Ethics oversight	Claire Johnson, MS, CPIA; 508-341-4271; Mispro IACUC committee

Note that full information on the approval of the study protocol must also be provided in the manuscript.

Plants

Seed stocks	N/A
Novel plant genotypes	N/A
Authentication	N/A
